# *Salmonella* Typhi and S*almonella* Paratyphi prevalence, antimicrobial susceptibility profile and factors associated with enteric fever infection in Bahir Dar, Ethiopia

**DOI:** 10.1038/s41598-021-86743-9

**Published:** 2021-04-01

**Authors:** Tadele Amsalu, Chalachew Genet, Yesuf Adem Siraj

**Affiliations:** 1 Diagnostic Medical Laboratory Unit, Dangila Primary Hospital, Dangila, Ethiopia; 2 Department of Medical Laboratory Science, College of Medicine and Health Science, Bahir Dar University, P. O. Box: 79, Bahir Dar, Ethiopia; 3 CDT-Africa, College of Health Sciences, Addis Ababa University, Addis Ababa, Ethiopia

**Keywords:** Microbiology, Health care

## Abstract

Enteric fever (EF) is caused by *Salmonella enterica* serovars Typhi (*S*. Typhi) and Paratyphi (*S*. Paratyphi) causing significant health problems in developing countries including Ethiopia**.** Thus present study aimed to determine prevalence and antimicrobial resistance profile of *S*. Typhi and *S*. Paratyphi among EF suspected patients at Felege-Hiwot comprehensive specialized hospital, Bahir Dar, Ethiopia. Hospital based cross-sectional study was conducted from March-to-May 2020. Totally, 150 patients were included conveniently. Data were collected using questionnaires by face-to-face interview. Concurrently, venous blood and stool specimens were collected and processed following standard bacteriological technique. Antimicrobial susceptibility test (AST) was performed by disc diffusion method. Logistic regression was performed to identify factors associated with EF infection. The study indicated 5.3% EF prevalence where *S*. Typhi accounted 75%. *S*. Typhi and *S*. Paratyphi isolates were 100% sensitive to cephalosporins but at least 83.3% showed resistance against chloramphenicol and tetracycline. At least 66.7% of isolates were multidrug resistance (MDR). Using well water for drinking (AOR = 6.22, CI 1.4–27.5) and previous EF history (AOR = 10.74, CI 2.01–55.9) were significantly associated with EF infection. Thus high bacterial prevalence and MDR isolates was observed. Therefore, health professionals should consider AST and use antibiotics with cautions for EF patient management.

## Introduction

Enteric fever is a faeco-orally transmitted bacterial disease comprising typhoid fever (TF) and paratyphoid fever (PTF) caused by *Salmonella enterica* serovars typhi (*S*. Typhi) and *Salmonella enterica* serovars paratyphi (*S*. Paratyphi) respectively^1,2^. Globally in 2017, EF caused 14.3 million cases and 135,900 deaths^3^ where 76.3% of the cases were caused by *S*.

Furthermore, TF causes about 11–20 million cases and 128,000 to 161,000 deaths as well as PTF cause 6 million cases and 54,000 deaths every year globally^4^. Though there is no licensed vaccine for PTF, the World Health Organization recommended the use of typhoid conjugative vaccine in 2017 for TF^3^. Despite a decrease in the morbidity and mortality associated with EF in industrialized countries, EF is one of major public health problems in sub-Saharan countries having 2.6 times more TF incidence than the overall incidence in low and middle income countries. Besides, because of low safe water access and sanitary facilities, EF is a common health problem in Ethiopia^2,3,5–7^. Furthermore, the impact of EF is more complicated because of an ever increasing emergency of antimicrobial resistance (AMR), including multidrug resistance (MDR), in *S.* Typhi and *S.* Paratyphi for commonly prescribed antibiotics^8–11^.

Depending on different factors such as clinical specimen and laboratory method used, the prevalence of EF is different globally ranging from 0.53 to 10.6%^12–17^. Previous studies in Ethiopia indicated a prevalence of 2.7–11%^18,19^. Furthermore different factors such as drinking unprotected or untreated water, eating unwashed foods, having unimproved or damaged sanitation facilities were associated with EF infection^1,13^.

Knowledge of local EF burden, AMR profile of *S*. Typhi and *S*. Paratyphi together with identifying risk factors for infection acquisition are essential in developing proper strategies for typhoid and paratyphoid fever prevention and control^4,7,20^ since there is considerable incidence variations in time and space^1,21^. Thus the present study was intended to determine the prevalence, antimicrobial susceptibility profile and factors associated with *S*. Typhi and *S*. Paratyphi infections among patients clinically suspected for EF at Felege-Hiwot comprehensive specialized hospital (FHCSH), Bahir Dar, Northwest Ethiopia.

## Methods

### Study area, study design and period

A hospital based cross sectional study was conducted at FHCSH, Bahir Dar, North West Ethiopia from March to May 2020 among patients clinically suspected as having enteric fever by attending physician. FHCSH, which was established in 1952, serves for more than 5 million people living in Bahir Dar city and surrounding zones. The hospital has 13 wards, 430 beds and about 531 health professionals. In the hospital patients clinically suspected as having EF are diagnosed using Widal test, the old serological test than culture. The present study will give the extent of culture confirmed EF prevalence in the study area.

### Inclusion and exclusion criteria

All patients having signs and symptoms of TF including fever (axillary temperature > 37.5 °C), abdominal pain or discomfort, headache, constipation or diarrhea and give written informed consent were included. On the other hand, patients clinically suspected as having EF but unconscious during the study period or on antibiotic treatment were excluded.

### Sample size and sampling technique

A total of 150 study participants were included using a single population proportion formula taking 11% prevalence of EF from previous studies in northwest Ethiopia^19^, 5% margin of error and 95% level of confidence. A convenient sampling method was used to select study participants until the required number was achieved.

### Data collection on demographic and other variables

Data were collected on demographic variables such as ages, sex, residence and other variables such as toilet availability, hand wash after toilet, water source, eating habit, history of EF and HIV serostatus using a pre-tested structured questionnaire using a face-to-face interview from patients clinically suspected for EF by attending physician.

### Clinical specimen collection

After the interview, venous blood and stool specimens were collected aseptically from each study participant. Ten ml and 3 ml blood specimens were collected aseptically from adults and children respectively using culture bottles with tryptone soya broth (Oxoid; Hampshire UK). Besides, fresh stool specimens were collected using sterile screw capped containers. Both specimens were transported to medical microbiology research laboratory of Bahir Dar University College of Medicine and Health Sciences within two hours of collection.

### Bacterial isolation and identification

Each blood culture bottle was incubated at 35 °C and observed daily for 7 consecutive days for microbial growth evidenced by presence of hemolysis, gas formation or media color change. But blood culture broth with no visual evidence of bacterial growth after 7 days of incubation was sub-cultured before it was considered as negative. Culture bottles which showed growth were opened aseptically and small amount of broth was taken using sterile wire loop and sub cultured into blood agar plate and MacConkey agar (BIOMARK Laboratories, India). The liquid stool specimen was processed directly but fecal suspension was prepared from formed stool specimen using normal saline. Four drops of fecal suspension was added into bottles containing selenite F broth (Oxoid; Hampshire UK) and incubated at 35 °C for 18 h and then sub-cultured into xylose-lysine-deoxycholate agar (XLD) (BIOMARK Laboratories, India), and MacConkey agar (BIOMARK Laboratories, India) further incubated at 35 °C for 24 h. Identification of *Salmonella* genus were done based on colony morphology, Gram staining and biochemical test following standard bacteriological methods. Further serovar identification of *S*. Typhi and *S*. Paratyphi were performed by automated microbiological technique using VITEK 2 system (BioMérieux diagnostics, France)^22^.

### Antibiotic susceptibility testing

Antibiotic susceptibility testing (AST) was done by using Kirby-Bauer disk diffusion method on Muller–Hinton agar (Oxoid, Hampshire UK) based on 2019 Clinical Laboratory Standard Institute (CLSI) guideline^23^. The turbidity of *S*. Typhi or *S*. Paratyphi isolates were adjusted using 0.5 McFarland standard prepared from barium sulphate. All isolates were tested against amoxicillin-clavulanate (20/10 µg), ceftriaxone (30 µg), cefotaxime (30 µg), cefoxitin (30 µg), ceftazidime (30 µg), gentamycin (10 µg), tetracycline (30 µg), ciprofloxacin (5 µg), nalidixic Acid (30 µg), chloramphenicol (30 µg) and cotrimoxazole (1.25/23.75 µg). Antibiotics used were selected based on CLSI guideline^23^, local prescription pattern, availability and all were Oxoid (Basingstoke, Hampshire, England). Results of AST were interpreted based on CLSI 2019 guideline^23^.

### Quality control

Before data collection, the questionnaire was pre-tested and every questionnaire was checked for completeness after collection. All culture media was prepared following manufacturer’s instruction. A sample of culture media plates prepared from each batch was incubated at 37 °C for 24 h to cheek for sterility. Before inoculation, the culture media was visually inspected for any microbial growth or deterioration. Moreover, McFarland standard was used to standardize inoculums density of bacterial suspension for the AST. Furthermore, *Salmonella* Typhomurium ATCC 14028 and *Escherichia coli* ATCC 25922 standard strains were used as quality control^23^. Furthermore all methods section including sample collection, bacterial isolation and AST were performed in accordance with CLSI and WHO guidelines^22,23^.

### Data analysis

Collected data were entered and analyzed using Statistical Package for Social Science 25 (IBM Corp Released 2011.IBM SPSS statistics. Armonk, NY: IBM Corp). Descriptive statistics was used to describe the demographic & other characteristics of the study participants, bacterial isolates and their AMR profile. Multivariable analysis was done to determine factors associated with EF infection and P-value < 0.05 was considered as statistically significant.

### Ethics approval and consent to participate

Ethical clearance was obtained from the Institutional Review Board (IRB) of Bahir Dar University, College of Medicine and Health Sciences protocol number 0013/2020. Moreover before data collection, written informed consent was obtained from each study participant with age greater than or equal to 18 years. Furthermore, from study participants with age less than 18 years, a written informed assent was obtained from their parents or legal guardian. In addition, all the information obtained from the study participants was registered by code to maintain confidentiality and culture positive results were communicated with responsible physician for proper patient management.

## Results

### Prevalence of enteric fever

Among 150 study participants, 81 (54%) and 95 (63.3%) were females and urban residents respectively. The age range of study participants were 8–80 with a mean of 34.1 and median of 32.5 age in years. The overall prevalence of culture confirmed enteric fever was 5.3%. Enteric fever was more prevalent on age group of 1–10 years (11.1%), females (6.2%) and rural residents (7.3%) than the other age groups, males and urban residents respectively. Moreover, enteric fever was more prevalent on participants who cannot read and write (7.4%) than participants who are educated (Table 1).

**Table 1:** Prevalence of enteric fever among different socio-demographic variables of study participants (n = 150) at FHCSH, Bahir Dar, Northwest Ethiopia, 2020.

Variables	CCEF: N (%)	EF negative: N (%)	Total EFSPP: N (%)
**Age (in years)**			
1–10	1 (11.1)	8 (88.9)	9 (6.0)
11–20	1 (5.3)	18 (94.7)	19 (12.7)
21–30	2 (5.6)	34 (94.4)	36 (24.0)
31–40	1 (2.8)	35 (97.2)	36 (24.0)
41–50	2 (7.1)	26 (92.9)	28 (18.7)
> 50	1 (4.5)	21 (95.5)	22 (14.6)
**Sex**			
Male	3 (4.3)	66 (95.7)	69 (46.0)
Female	5 (6.2)	76 (93.8)	81 (54.0)
**Education**			
Can’t read & write	4 (7.4)	50 (92.6)	54 (36.0)
Primary education	1 (4.8)	20 (95.2)	21 (14.0)
Secondary education	1 (2.9)	33 (97.1)	34 (22.7)
Diploma and above	2 (4.9)	39 (95.1)	41 (27.3)
**Occupation**			
Civil servant	1 (3.3)	29 (96.7)	30 (20.0)
Merchant	1 (4.3)	22 (95.7)	23 (15.3)
Farmer	2 (6.2)	30 (93.8)	32 (21.4)
Daily laborer	1 (8.3)	11 (91.7)	12 (8.0)
Housewife	2 (8.7)	21 (91.3)	23 (15.3)
Student	1 (3.3)	29 (96.7)	30 (20.0)
**Residence**			
Urban	4 (4.2)	91 (95.8)	95 (63.3)
Rural	4 (7.3)	51 (92.7)	55 (36.7)
Total	8 (5.3)	142 (94.7)	150 (100)

*CCEF* culture confirmed enteric fever, *N* number, *EFSPP* enteric fever suspected patients processed.

**Figure 1:**
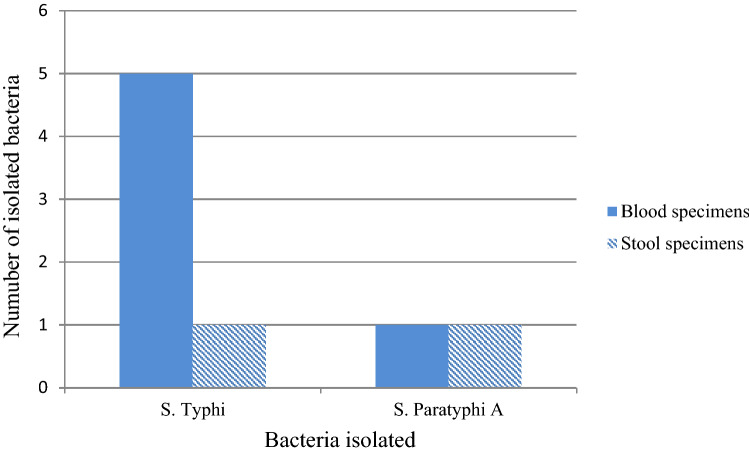
Distribution of *Salmonella* serovars among different clinical specimens obtained from study participants (n = 150) attending at FHCSH, Bahir Dar, Northwest Ethiopia, 2020.

In the present study, the prevalence of TF (4%) was higher than PTF (1.3%). From the total 8 culture confirmed EF patients, 75% was caused by *S*. Typhi and 25% by *S*. Paratyphi A with no co-infection. Besides, 75% and 25% of *Salmonella* species were isolated from blood and stool specimens respectively (Fig. 1).

### Antimicrobial resistant profile of *S. *Typhi and* S.* Paratyphi isolates

*S*. Typhi revealed the highest resistance rate for tetracycline and chloramphenicol with 83.3% for each. Similarly, all *S*. Paratyphi A isolates were resistant for tetracycline, chloramphenicol and amoxicillin-clavulanate. On the other hand, all *S*. Typhi and *S*. Paratyphi A isolated were susceptible for cefotaxime, ceftazidime and cefoxitin (Table 2). Furthermore, 4 (66.7%) and all isolates of *S*. Typhi and *S*. Paratyphi A were multidrug resistance (MDR) respectively.

**Table 2:** Antimicrobial resistance pattern of *S.* Typhi and *S*. Paratyphi A isolated from study participants (n = 150) attending at FHSCH, Bahir Dar, Northwest Ethiopia, 2020.

Antibiotics tested	Antimicrobial resistance profile of isolates					
	*S.* Typhi (n = 6)			*S.* Paratyphi A (n = 2)		
	R: N (%)	I: N (%)	S: N (%)	R: N (%)	I: N (%)	S: N (%)
Amoxicillin-clavulanate	4 (66.6)	1 (16.7)	1 (16.7)	2 (100)	0 (0)	0 (0)
Gentamycin	1 (16.7)	1 (16.7)	4 (66.6)	1 (50)	0 (0)	1 (50)
Tetracycline	5 (83.3)	1 (16.7)	0 (0)	2 (100)	0 (0)	0 (0)
Ciprofloxacin	1 (16.7)	0 (0)	5 (83.3)	0 (0)	1 (50)	1 (50)
Nalidixic acid	3 (50)	2 (33.3)	1 (16.7)	0 (0)	0 (0)	2 (100)
Chloramphenicol	5 (83.3)	1 (16.7)	0 (0)	2 (100)	0 (0)	0 (0)
Cotrimoxazole	3 (50)	1 (16.7)	2 (33.3)	1 (50)	1 (50)	0 (0)
Ceftriaxone	1 (16.7)	0 (0)	5 (83.3)	1 (50)	0 (0)	1 (50)
Cefotaxime	0 (0)	0 (0)	6 (100)	0 (0)	0 (0)	2 (100)
Cefoxitin	0 (0)	0 (0)	6 (100)	0 (0)	0 (0)	2 (100)
Ceftazidime	0 (0)	0 (0)	6 (100)	0 (0)	0 (0)	2 (100)

*R* resistant, *I* Intermediate, *S* Sensitive *N* number of isolates.

### Multivariable analysis on risk factors of EF infection

Based on multivariable analysis, using well water for drinking and a previous history of EF were significantly associated explanatory factors for enteric fever infection. Patients who have previous history of EF infection and used well water for drinking had 16.4 and 14.9 times more chance of developing EF than those who didn’t have a history of EF infection and used pipe water for drinking respectively. Though it was not significant (P = 0.365), a considerable difference in EF infection was observed among participants who didn’t have toilet (9.5%) than those having toilet (4.7%). Despite higher EF infection compared with their counter parts, consuming raw meat (P = 0.402), raw vegetable (P = 0.510), street food (P = 0.573) and drinking raw milk (P = 0.569) was not significantly associated with EF infection (Table 3).

**Table 3:** Factors associated with prevalence of enteric fever among febrile patients (n = 150) at FHCSH, Bahir Dar, Northwest Ethiopia, 2020.

Variable	Total: N (%)	Positive: N (%)	COR	AOR	P-value
**History of EF**					
Yes	35 (23.3)	5 (14.3)	6.22 (1.4–27.5)	16.4 (1.7–161.5)	0.02
No	115 (76.7)	3 (2.6)	1	1	
**Toilet usage**					
Yes	98 (65.3)	2 (2.0)	1	1	
Sometimes	42 (28.0)	4 (9.5)	5.05 (0.88–28.7)	1.25 (0.10–14.6)	0.68
No	10 (6.7)	2 (20.0)	12.0 (1.48–96.8)	4.73 (0.24–91.8)	0.30
**Hand wash after toilet**					
Yes	82 (54.7)	2 (2.4)	1	1	
No	68 (45.3)	6 (8.8)	3.87 (0.75–19.8)	0.87 (0.08–9.93)	0.51
**Soap use**					
Yes	85 (56.7)	1 (1.2)	1	1	
No	65 (43.3)	7 (10.8)	10.0 (1.125–84)	11.0 (0.90–134)	0.07
**Water source**					
Pipe water	112 (74.7)	1 (0.9)	1	1	
Well water	38 (25.3)	7 (18.4)	10.74 (2.01–55.9)	14.9 (1.4–162.4)	0.01
**HIV serostatus**					
Positive	12 (8.0)	3 (25.0)	8.87 (1.82–43.1)	0.29 (0.03–3.29)	0.39
Negative/unknown	138 (92.0)	5 (3.6)	1	1	

*EF* enteric fever.

## Discussion

Enteric fever is a widespread public health problem in low and middle income countries including Ethiopia^21,24^, where 88% urban and 92% rural residents don’t treat drinking water and only 6% of households use improved toilet facilities^25^. In the present study, the culture confirmed prevalence of EF was 5.3%. This was comparable with previous studies in Ethiopia^26^ and Bangladesh^16^ which reported 4.1% and 5% respectively. Similarly the prevalence of TF (4%) in the present study was comparable with previous studies in Shashemene, Ethiopia^27^ and abroad in Egypt^28^. But the prevalence of EF in the present study was higher than a study done in Ethiopia^18^, India^12,29,30^, Fiji^13^, and Nepal^15^ reporting 2.7%, 0.22–1.24%, 0.7%, and 2.5% respectively. Similarly lower TF prevalence than the present study was reported by previous studies done in Cameroon (2.5%), Asian countries (2%), India (0.14%), and Laos (1.5%)^29,31–33^. This could be due to variations in *S*. Typhi and *S*. Paratyphi prevalence in place, time and even in consecutive years at the same geographical location^21^ and seasonal variability^34^. Furthermore sensitivity differences in the laboratory detection methods used might also contribute for the difference observed.

In contrast to our findings, previous studies in Jigjiga, Ethiopia^19^, Nepal^14,35^, India^17^, Nigeria^36^ and Indonesia^37^ reported a higher EF prevalence among febrile patients ranging from 11 to 14.1%. This might be due to the varied incidence of TF in different study areas and periods^38^. Moreover, the geographical heterogeneous nature of TF burden^21,32^ might also contribute for the difference observed. Furthermore, higher prevalence of *S*. Typhi than *S*. Paratyphi was documented in the present study. Previous studies have reported comparably higher TF than PTF prevalence in Ethiopia^19,26^, Indonesia^37^, Bangladesh^16^, India^12^, Fiji^13^, Nepal^14^, India^17,30^, and Indonesia^37^. On the other hand, few studies in Ethiopia and abroad reported higher prevalence of *S*. Paratyphi than *S*. Typhi^18,36^. In a meta-analysis done on EF^24^, *S*. Typhi accounted 76.1% which might indicate its higher share in causing EF than *S*. Paratyphi.

Though higher EF prevalence in patients who consumed street foods was documented in the present study, it was not significant. But a significant association was reported from studies in Indonesia^37^. The difference might be due to differences in study design and area. Furthermore, there may be variations on street food type and extent of cooking. On the other hand, using well water for drinking and previous history of EF were significantly associated explanatory factors for EF infection. Comparable findings were reported from previous studies in Ethiopia^19,27^. The significant association of previous history of EF with current EF infection might be due to the reactivation from previous infections.

Antimicrobial resistance in *S*. Typhi and *S*. Paratyphi in low and middle-income countries is worsening^24^. Despite the establishment of AMR surveillance in Ethiopia^39^, it is a growing challenge where the overall prevalence of MDR is high and inappropriate antibiotic use such as self-medication is common^40^. In the present study, *S*. Typhi and *S*. Paratyphi A showed variable resistance level to different categories of antibiotics tested. Both *S*. Typhi and *S*. Paratyphi showed the highest resistance level against tetracycline and chloramphenicol with more than 83% for each. The resistance level for chloramphenicol in both isolated bacteria in the present study was comparable with previous studies^19^ but higher than previous reports for *S*. Typhi^16,28,30,33,36,41,42^ and *S*. Paratyphi^16,36,42^. Furthermore the resistance level against tetracycline for both bacteria was comparable with study in Nigeria^36^ but higher than study in Ethiopia^19^. Similarly a significantly higher resistance level of *S*. Typhi and *S*. Paratyphi A was documented against tetracycline and chloramphenicol in the present study than study in Nepal (13.56%)^35^. Furthermore, half of *S*. Typhi and *S*. Paratyphi A isolates in the present study were resistant for cotrimoxazole which was comparable with studies in Nigeria^36^, and Laos^33^ but higher than study in India^30,42^. This variation might be due to study period and setting difference. Besides this, increased resistance level from year-to-year for different antibiotics such as chloramphenicol and cotrimoxazole^42,43^ might be a contributing factor.

Half of *S*. Typhi and both of *S*. Paratyphi A isolates were susceptible to nalidixic acid. The resistance level of *S*. Typhi against nalidixic acid in the present study was comparable with a study in India^42^. Though there was a study which reported higher resistance level^35^, many previous studies documented lower resistance level of *S*. Typhi against nalidixic acid than the present study^16,19,41^. Furthermore, 83.3% of *S*. Typhi and 50% of *S*. Paratyphi A isolates were susceptible to ciprofloxacin. In the present study, the resistance level of *S*. Typhi against ciprofloxacin was comparable with previous studies^16,19,41^ but higher resistance level of *S*. Paratyphi A was documented than previous studies^16,19^. The resistance level for both bacteria in the present study was higher than studies in Nepal^35^, Nigeria^36^, and India^42^. This increased fluoroquinolone resistance was also supported by previous study reporting an increased level of resistance by *S*. Typhi and *S*. paratyphi over years^14,43^.

On the other hand, all isolates of *S*. Typhi and *S*. Paratyphi A were susceptible to cephalosporins including cefotaxime, cefoxitin and ceftazidime. Unlike *S*. Typhi which revealed 100% sensitivity, half of *S*. Paratyphi A showed resistance to ceftriaxone. Comparable susceptible result was reported for *S*. Typhi in previous studies for ceftriaxone^16,19,35,36,42^ and ceftazidime^36^. The resistance level in the present study was higher than previous study reports for *S*. Paratyphi A against ceftriaxone^16,19,35,36,42^. But *S*. Paratyphi A showed lower resistance to ceftazidime than previous reports^36^. The increased resistance of *S*. Typhi and *S*. Paratyphi A in the present study against different classes of antibiotics might be due to sample size difference, antibiotic misuse, and inappropriate prescribing practice of health professionals coupled with resistance gene transfer among different *Salmonella* species.

The present study revealed that 66.7% of *S*. Typhi and all isolates of *S*. Paratyphi were MDR. Though the MDR level of *S*. Typhi in the present study was comparable with a study in Bangladesh (64.28%)^41^, it was higher than many previous studies in Ethiopia^19^, and elsewhere such as Egypt, Nigeria, Asian countries, India, and Laos^28,32,33,36,42^ which reported 0 to 29%. Similarly the MDR level of *S*. Paratyphi in the present study was higher than studies in Ethiopia^19^ and abroad^24,36,42^ which documented 0 to 25%. The high MDR level was supported by meta-analysis study which indicated the worsening AMR trend among S. Typhi and S. Paratyphi^24^. The current study is limited in that it has low sample size and did not isolate resistance genes from both S. Typhi and S. Paratyphi isolates.

## Conclusions

High prevalence of EF mainly caused by *S*. Typhi was observed. Besides increased resistance of *S*. Typhi and *S*. Paratyphi for commonly prescribed antibiotics in the study area such as ciprofloxacin and ceftriaxone were revealed than previous reports. Besides, the majority of *S*. Typhi and all isolates of *S*. Paratyphi A were MDR. On the other hand all *S*. Typhi and *S*. Paratyphi A isolates were fully susceptible to cefoxitin and ceftazidime. Besides previous history of EF and using well water for drinking were significantly associated explanatory factors with EF infection. Thus treatment of EF should be supported with AST mainly in patients having a history of EF infection. Health professionals should use antibiotics with cautions during empirical therapy of EF suspected patients. Including EF topics on their health education system will be helpful. Besides, further large scale and molecular studies are recommended to understand the extent of *S*. Typhi and *S*. Paratyphi prevalence, their AMR profile and reveal AMR genes.

## Data Availability

All relevant data are included within the manuscript.

## Abbreviations

AMR: Antimicrobial resistance
AST: Antimicrobial susceptibility test
CLSI: Clinical Laboratory Standards Institute
EF: Enteric fever
FHSCH: Felege–Hiwot comprehensive specialized hospital
IRB: Institutional review board
MDR: Multidrug resistance
PTF: Paratyphoid fever
TF: Typhoid fever
WHO: World Health Organization
